# Hospital and Institutional News

**Published:** 1919-02-01

**Authors:** 


### HOSPITAL AND INSTITUTIONAL NEWS.

### STUDENTS DISCUSS STATE MEDICAL SERVICE.

"St. Mary's Hospital Gazette," as well as very complete records of St. Mary's Hospital news, usually succeeds in publishing, month by month, something of interest to those outside its family circle. In the January number the *Gazette* reproduces in full a paper on "The Necessity for a Public Medical Service," read at a meeting of St. Mary's Hospital Medical Society by Mr. C. A. Parker, F.R.C.S. (Edin.). It is very desirable that such papers should be read before and discussed in hospital societies, for it is well that all students should be well informed on the subject, which is of growing importance and which may have great effect on their future. Mr. Parker has probably studied "A Vision of State Medical Service" which was recently published in The Hospital, and can now be obtained in pamphlet form from The Scientific Press. Whether he has learnt from that pamphlet or no, the scheme in it has been adopted in great part in his paper. Mr. Parker clearly sees that the chief reason a State Medical Service is adumbrated is that the public is not satisfied that it is getting all it should out of the knowledge the research of the last decades has put at the disposal of medical men. He thinks that individualistic private practice has failed to provide the best of treatment, and that it never will be able to provide it. It would be interesting to know how far the meeting approved the proposals. Perhaps some St. Mary's man will write and let us know.

### THE FUTURE OF GENERAL PRACTICE.

On January 14, at 11 Chandos Street, the London Association of Medical Women also discussed State Medical Service. On the whole, the meeting seems to have acknowledged the desirability of some scheme, though from the report of the proceedings it does not seem to have committed itself to any detail. The principles of hospital treatment, when desirable, for all patients, and study-leave at intervals for all medical practitioners, were agreed to. It seems as if the medical profession is acknowledging the necessity of, or resigning itself to the prospect of, State Medical Service.

### THE SWINEY PRIZE FOR 1919.

The Swiney Prize for 1919 has been awarded to Dr. Mercier. The prize is a silver-gilt cup designed by Maclise, and of the value of £100, and money to the same amount. It is awarded every fifth year to the writer of the best work on jurisprudence published since the last award, but, as it is given alternately for general jurisprudence and for medical jurisprudence, the period of each award is practically ten years. The prize was founded in 1844, and in the seventy-five years of its existence has been awarded to very distinguished men, including Professor Leone Levi, Dr. A. S. Taylor, Sir Henry Maine, Sir Robert Phillimore, Professor Sheldon Amos, Sir F. Pollock, and Professor Maitland. Dr. Mercier won it in 1909, and this is the first occasion on which it has been awarded to the same candidate for the second time. The book that has won the prize this year is entitled "Crime and Criminals," published in December 1918.

### HOSPITAL PHARMACISTS AND TRADE UNIONISM.

The hospital pharmacists of London and district have been invited by the Amalgamated Society of Drug and Chemical Workers to form a "section" in that Union. A propaganda meeting held recently was addressed by Mr. H. Skinner, pharmacist, Great Northern Hospital (President of the Union), who urged that the ordinary associations could not act with such effectiveness as trade unions on questions of salary. Many questions were asked. The majority of those present agreed that, in the event of extreme measures being taken by other sections of the Union, it would be fatal to hospital pharmacists not to join in. Mr. F. A. Hocking (London Hospital) disagrees with the Union named, principally because it contains a large number of the non-professional element, such as drug porters, unqualified assistants, and even carmen who deliver goods at hospitals. Mr. Hocking advised hospital pharmacists to organise themselves on a professional basis, following the example of medical men. Mr. Hocking's suggestion, it may be added, is partly in being already, as the Public Pharmacists' Association is restricted to duly qualified pharmacists. Besides hospital pharmacists, however, pharmacists engaged at infirmaries, mental hospitals, etc., are eligible for membership. It will be interesting to note the result of the movement initiated by Mr. Skinner, and whether Mr. Hocking's proposals will lead to an accession of strength to the Public Pharmacists' Association.

### A NORTHAMPTON RESIGNATION.

Dr. G. H. Percival, senior honorary surgeon at the Northampton General Hospital, has resigned. He was appointed house surgeon as long ago as 1878 and has done much admirable work for the Institution the operations of which have been greatly extended since he first became associated with it. Dr. Percival sent in his resignation at the beginning of the war, but owing to the exigencies of the situation he consented to withdraw it for the time being, and he has with characteristic self-sacrifice continued to carry on the duties. The Board of Management, in receiving the resignation with much regret, expressed the highest appreciation of Dr. Percival's services. It may be mentioned that the name of Percival has for very many years been held in high honour in Northampton; and Dr. Percival's father carried on the good work before him.

### PRESENTATION TO MR. GLENTON KERR.

Lord William Cecil, the chairman of the Queen's Hospital for Children, Hackney Road, presented a testimonial and a cheque to Mr. Thomas Glenton Kerr to commemorate his twenty-five years' service. After speeches had been made by the chairman, Mr. Joseph Meller, the chairman of the House Committee, and Sir Arthur Spurgeon, chairman of the Little Folks' Home at Bexhill, Mr. Glenton Kerr thanked the donors for their "beautiful memorial," and continued: "These twenty-five years have been years of hard work but of happiness too through the kindness and keenness of my committee. This particular hospital's work is now seen to be the head and front of this class of work in London. But we have a great task ahead, as Lord William Cecil hinted, and that is the completion of the Committee's building scheme. That scheme has had to be postponed since the beginning of the war, but two recent events are encouraging. The first jubilee of the hospital, though actually it took place two years ago, gives an admirable opportunity for new effort, and secondly King Edward's Hospital Fund has set aside £1,250 on its own initiative to start a fund to build an out-patient department for the hospital." The testimonial was arranged by Mr. Joseph Meller. We give its text in the following note:

### "1893—1918.

"Presented to Mr. Thomas Glenton Kerr on the completion of twenty-five years, as secretary of the Queen's Hospital for Children, Hackney Road, London, in appreciation of his unsparing industry in the cause of the institution, his kindly thought and consideration for all his fellow workers, and his brave and cheerful hopefulness in times of difficulty." The fact that the hospital has during his time of office grown from 57 beds to 164 beds, that the annual revenue has been increased from an average of £5,500 to £20,000, and that no less a sum than £73,000 has been collected for enlargement and improvement bears eloquent testimony to his zeal and energy. The signatures are Shaftesbury, William Cecil, M. Ruffer, Joseph Meller, and the testimonial is dated January 23, 1919. Any review of hospital developments which began before the foundation of King Edward's Fund gathers within its purview immense changes. The voluntary system survives, but the very word system which is now in common use marks the change which has taken place from the private philanthropy of one hundred years ago, to the great but free organisations of the present. It has been the growth of this development which Mr. Kerr has been privileged to participate in.

### HOTELS FOR BABIES.

The day nursery we know; and the crèche, which is the same thing under a more fashionable name, has become familiar. Now His Majesty the Baby is to have his own hotels. The innovation is an indication of the trend of the times, and a proof of the growing interest taken in child life and welfare. Two establishments are to be opened in Clissold Park, Stoke Newington, London, under the auspices of the National League for Health, Maternity, and Child Welfare, whose headquarters are 5 Tavistock Square, W.C. The American Red Cross Society has given £1,500 towards the cost of the object, and the British Red Cross is to supply new furniture. One of the "hotels" is for the children up to seven years of age of the professional classes, for whom there will be a charge of one to two guineas a week. Mothers who happen to be abroad, or others who are moving about, such as actresses, will here find safe custody and protective care for their offspring. The second "hotel" is for babies of the working classes, and the charge will be 10s. per week, any loss being made up by contributions, for which an appeal is made. This establishment is expected to solve the problem of mothers who may be in hospital, or of fathers who are left with motherless children. The matron of the "hotels" is Miss Hodge, late of the East London Hospital for Children, who will be assisted by an expert staff. The experiment is an interesting one, and seems to supply a connecting link that has long been missing.

### THE IPSWICH WAR MEMORIAL.

A big scheme is being prepared in connection with the East Suffolk and Ipswich Hospital, which will probably be the County War Memorial. It aims at completing the reconstruction, which was begun some years ago, by the erection of further special wards and special departments. The department of bacteriology would be extended so that more research might be undertaken, and in addition to new buildings two motor ambulances would be acquired. A new convalescent home is projected at Felixstowe, to which the patients could be carried by means of the motor cars, and at the same time the entire area served by the hospital would be brought into touch with it. An appeal is being issued which gives the details of the scheme—the latest step in the expansive policy of recent years.

### FALLACIES ABOUT ILLEGITIMATES.

Reporting to the Durham County Council, the Superintendent Health Visitor refers to the great need for institutions for illegitimate babies, urging that it is better for mothers and babies to be together, at least during the child's first year. But, she proceeds to point out, all mothers of illegitimate babies are not young girls more sinned against than sinning, and willing to be helped along orthodox lines. There are many mothers who refuse to go into homes, and whose object appears to be to get rid of their babies, anyhow, as quickly as possible. Few people realise the ghastly cruelties that are being practised upon unwanted babies in England to-day. The Superintendent believes that with better methods of collecting maintenance under affiliation orders and enforced contributions from the mother according to her earning capacity, the careless and thoughtless might come to realise the public aspect of their behaviour.

### HEALTH VISITORS AND THE EPIDEMIC.

In her report to the Durham County Council the Superintendent Health Visitor also indicates the lesson which the epidemic provides. The health visitors, she says, did yeoman service in many parts of the county. Some of them worked day and night, on weekdays and Sundays. They nursed the people, made bread, and even butter in one farmhouse emergency. They also tried to organise home helps schemes, but, in most instances, with little success, because the epidemic was so sudden that the worst was over before a start could be made. At Southwick, the school teachers came to the rescue and started an invalid food depôt in co-operation with the county health visitor. The funds were provided by the District Council. There was no one to prepare invalid food, or, indeed, any kind of food. In some cases the delirious and the dying, left unattended, wandered into the streets in their night clothes.

### APATHY AND DISTRICT NURSES.

One great lesson to be learned from the epidemic is the importance of completing the county nursing scheme, so that every area may have its nurse and the whole county a reserve of nurses for emergencies. It is difficult to understand the apathy with which the establishment of district nurses is regarded in many parts of the county. It cannot be a question of money, for the total expenses of a nurse would not be more than £3, that is, 720 pennies weekly. In most of our large villages more than that is spent in one night at the local picture house. The services of the local district nurse have often been found useful in emergencies in wealthy districts. Smaller areas would not be able to raise £3 weekly, but here nurse-midwives would be employed, part of whose salary would be recoverable in midwifery fees. Grants from the County Council for the nursing of certain diseases are available in all areas. This will not be the last epidemic, and trained workers cannot be produced at a moment's notice.

### A GUARANTEE FOR MIDWIVES.

Once more the Durham County Midwives' Federation—in the quarterly report for the period ended December 31—insists upon the need for more midwives, and mentions that in many districts this need must shortly become more pressing because there are now twenty-two practising midwives who are over 65 years of age. To supply this growing demand for midwives with the best type of woman for the work, it is imperative to guarantee them the chance of making a decent livelihood, otherwise they will enter other fields of nursing.

### HOME HELPS AND COTTAGE NURSING.

During 1919 the Durham County Council hopes to organise a Home Helps scheme. It is not the business of the district nurse to scrub, clean, cook, and do the family washing. When the mother in a cottage home is ill all these things must be done and often no suitable person can be found to do them, even when payment is guaranteed. Definite steps must be taken to tackle this problem.

### DEATH OF A GLAMORGAN BENEFACTOR.

On January 20th there died, after five days' illness, Lt.-Colonel John Arthur Jones, High Sheriff for Glamorgan, a generous benefactor of all good objects in the district. Although one of the busiest men in the commercial world, he found time for much public work and philanthropic efforts. He was, among other activities, a member of the Executive Committee of the Prince of Wales's Hospital for Limbless Sailors and Soldiers, Chairman of the Finance Committee for the Welsh War Hospital, Netley, hon. treasurer for the Queen Victoria Jubilee Nurses, Cardiff, and a member of the Board of Management of King Edward's Hospital, Cardiff. A few months ago he endowed a bed at the Prince of Wales's Hospital in memory of his only son, Lieut. Leslie Gwynne Jones, who was killed in France in May last. The death of his son undoubtedly affected Colonel Jones's health, but he continued his public work with a rare courage.

### DR. T. STACEY WILSON RETIRES.

Dr. T. Stacey Wilson has retired from the post of honorary physician to Birmingham General Hospital after 29 years' service. To commemorate his work he has been presented with an illuminated address. In acknowledging the gift he said that he was proud of his association of 34 years with the hospital, but the pathological laboratories were still insufficient and unworthy of the work to be done. A portrait of Dr. Wilson by Mr. Edward S. Harper was accepted by the president and will be hung in the board room. Dr. Wilson has held in turn all the important offices in the hospital, and the retiring age still leaves him in vigour and keenness.

### THE FUTURE OF ST. LUKE'S, BRADFORD.

At a meeting of representatives of the principal towns of Yorkshire to compare notes in regard to the shortage of hospital accommodation during the recent influenza epidemic, it was found that this shortage was general. In reference to the handing back to the Bradford Corporation of hospitals taken during the war, Mr. R. S. Dawson, chairman of the Guardians, referred to the future of S. Luke's. Officially, he said, nothing had been done. The proposal that the Corporation should take S. Luke's might have been considered by the Local Government Board, and if the proposed Ministry of Health were created, S. Luke's would pass to the Corporation and become a general municipal hospital. At present there was surplus accommodation there. Under the Act of 1875 the Health Committee had power to provide all the accommodation needed by the city, and Mr. Dawson said that there was no reason why any one should wait for accommodation. As it was, hundreds of people had died in Bradford for want of it.

### NEW NAMES FOR HOSPITAL BEDS.

Lancaster workpeople have handed to the Infirmary £2,474, the largest amount in the thirty years' history of the Committee. In 1917 the amount raised was £1,908, and that was the first year that the Committee had succeeded in giving over £1,000 a year to the Infirmary. They had been striving to this end for over a quarter of a century, and it is remarkable that the best results were obtained during the last two years of the war. The presence of a national factory near Lancaster has helped the funds materially during the past two years. The General Committee of the institution "named" one of the beds "The Workpeople's Bed," to mark the attainment of "a thousand a year for the Infirmary," and on the plate are inscribed the names of four original members of the Committee, who have worked amid many changes up to November 1917, when the secretary, Mr. Prescott, died. The three survivors still continue their services, one as chairman and acting secretary, another as treasurer, and a third as the chairman of one of the sub-committees. Another bed was "named" by the General Committee at the end of the year "The Projectile Bed," in appreciation of the fact that the workers and staff of the National Projectile Factory had contributed just over £3,000 in three years to the Infirmary funds. The names of the Projectile Hospital Committee are recorded on the inscribed plate.

### THE FUTURE OF RED CROSS HOSPITALS.

A concise and forcible appeal has been issued by the East Lancashire Branch of the British Red Cross Society The arguments on behalf of the appeal are of general interest. By the end of March, the writers point out, at least half these hospitals will no longer be required, and in June most of these will be closed. But there will still be needed special surgical hospitals and hospitals for limbless cases, both for several years, while a hospital for consumptives is already a necessity. There is, too, a large class of discharged men in need of treatment which is ineligible for admission to homes for the disabled. The committee feels these hospitals cannot be maintained any longer by divisional effort, but must be supported by the county fund. Subscriptions are likely to diminish, and for these reasons an appeal is made for the accumulation of a large reserve, in the early months of the present year, so that the future claims of the men may not be neglected. The voluntary hospitals, of course, are already helping discharged men, but they have now arrears of civilian cases to attend to, and the county funds should last so long as a single case for which the funds were created remains in need of hospital care.

### A FLAT RATE FOR SCHOOL CASES.

Some time ago the Kent County Council appointed representatives to meet doctors to discuss the charges for the treatment of school children at hospitals. The Council has now accepted the following charges which were proposed by the doctors: £1 1s. for all in-patients, £1 1s. for all out-patients requiring operations under general anæsthesia, and 7s. 6d. for all other patients. These fees will be payable to the doctors, but in some cases application may be made for a payment to the hospital which provides the accommodation. The doctors, on this point, expressed the opinion that in cases coming within the scale of £1 1s. a fee of 5s. would be sufficient for each child kept in hospital for one night or more, and 2s. 6d. for each other case, but eventually it was held desirable to fix a flat rate of 3s. 6d. per case.

### PATIENTS IN LODGINGS: A DOUBLE GRIEVANCE.

The methods of the Ministry of Pensions come in for severe criticism in a report of the West Riding War Pensions Committee. Numbers of men continue to be discharged from the Army, and there is a great dearth of accommodation for their treatment. The Committee some time ago suggested to the Ministry of Pensions that centres for orthopædic treatment should be established at the various civil hospitals in the Riding and that the Ministry should provide the necessary apparatus. So far no such provision has been made. At Harrogate a number of patients are still unable to obtain admission to a hospital and have to go into lodgings, in consequence of which the Ministry states that it is unable to give the allowances which would be payable to the men if inmates of a hospital. The Disablements Sub-Committee has declared that it will resign unless a remedy is promptly found.

### CITY CORONER'S WORK DURING 1918.

Dr. Waldo, H.M. Coronor for the City of London and Southwark, at a recent inquest, said: "Certain figures, in his return to the Home Secretary for 1918, were now on record, *inter alia*: he had altogether held 524 inquiries; and nearly all the deaths investigated were due to violence or unnatural causes, mainly of those dying in hospitals and institutions, and in the streets, or away from home. In these cases, in which the question of conduct and neglect were likely to arise, he had continued the pre-war practice of sitting with a jury, and he believed such a procedure gave greater satisfaction generally to the public, including the relatives of deceased persons, and of other interested parties. In short, he was of opinion that a jury, assisted by a coroner, is a far better tribunal for the elucidation of truth than a Coroner unassisted by a jury. There had been a decrease in the number of deaths caused by bombs dropped from enemy aircraft, the number being seven, compared with seventy-seven in 1917. On two occasions—including one sitting, when without warning, the Court being filled with women and children, seven men were killed close by—the City Coroner's Court had narrowly escaped demolition from this cause. The reduction in the number of fires was largely attributable to the present shortage of matches.

### THIS WEEK'S DRUG MARKET.

The market is still in a stagnant condition and the general trend of prices continues to be downward, although in a large number of cases these are merely nominal. Saccharin is selling at extraordinarily low figures, and cannot, one would think, recede much further. Sugar of milk is also selling at much lower prices. Menthol is inactive. Japanese refined camphor is lower, but the value of English refined is maintained. Phenazone, phenacetin, hexamine, paraldehyde, salicylic acid, all have an easier price tendency. Eucalyptus oil is in fairly good supply while the demand is less acute: the result is that the price is barely maintained.

